# How Bitter Medicine Could Clear Up Asthma

**DOI:** 10.1371/journal.pbio.1001500

**Published:** 2013-03-05

**Authors:** Janelle Weaver

**Affiliations:** Freelance Science Writer, Carbondale, Colorado, United States of America

[Fig pbio-1001500-g001]Airway obstructive diseases, such as asthma and chronic obstructive pulmonary disease, cause the airways to narrow and make it difficult to breathe. Broncodilators are used to treat these conditions, but they often don't work in severe cases and can cause serious side effects, such as abnormal heart rhythms and increased blood pressure. Recently, scientists discovered a potential alternative to currently available bronchodilators. These compounds act on bitter taste receptors in the airways called TAS2Rs, a class of proteins long thought to be restricted to taste buds on the tongue for the purpose of detecting and avoiding harmful toxins. Bitter substances are more effective than existing bronchodilators at causing the relaxation of smooth muscle cells—which control the diameter of the airways—and opening up the airways in a mouse model of asthma. But the mechanism of action of these promising compounds has been under dispute, limiting the potential of developing them into a new class of drugs for patients.

**Figure pbio-1001500-g001:**
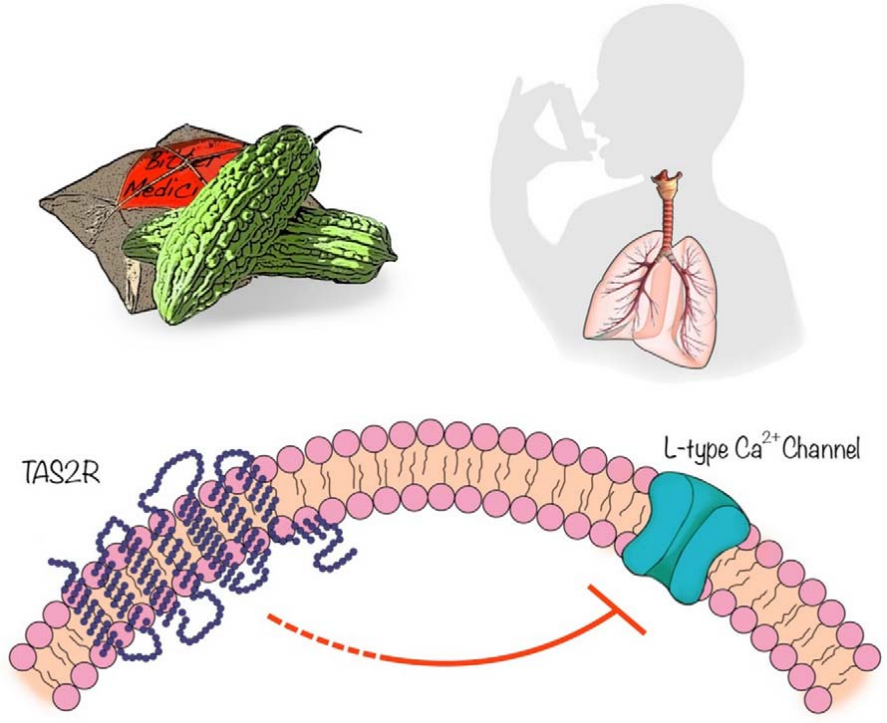
Bitter tasting compounds, synthesized or natively present in plants such as bitter melon, act on bitter taste receptors (TAS2R) in airway smooth muscle cells, which can inhibit L-type calcium channels and lead to muscle cell relaxation. **This action may help in the treatment of asthma.** Image credit: Qi Xiao, Nanjing University.

In this issue of *PLOS Biology*, a team led by Ronghua ZhuGe of the University of Massachusetts Medical School has uncovered how bitter compounds open up the airways. They found that these substances stimulate TAS2Rs to affect calcium signaling and contraction in smooth muscle cells. The study reveals a new cell-based screening method for quickly identifying bitter compounds that have the potential of becoming powerful broncodilators for the treatment of airway obstructive diseases.

ZhuGe and his team used freshly dissected smooth muscle cells and tissues, which should more accurately reflect calcium signaling and cell contraction than the cultured cells used in another recent study. Similar to the previous study, ZhuGe and his collaborators found that bitter compounds produced a counterintuitive effect: they stimulated TAS2Rs to cause a rise in calcium levels in smooth muscle cells, which might be expected to cause these cells to contract because broncoconstrictors also increase calcium levels in these cells. But unlike the previous study, the researchers discovered that the rise in calcium was not sufficient to cause the cells to contract.

Moreover, bitter compounds actually reversed the effects of broncoconstrictors by inhibiting L-type voltage-dependent calcium channels, thereby blocking calcium influx into smooth muscle cells and causing them to relax. Thus, bitter substances stimulate TAS2Rs to activate two opposing calcium signaling pathways, depending on the circumstances. Under normal conditions, the compounds can modestly raise calcium levels in smooth muscle cells without causing contraction, but they reverse the rise in calcium levels caused by broncoconstrictors. Together, these findings resolve the paradox about how bitter substances can cause airway dilation even though they raise calcium levels in smooth muscle cells.

By revealing the mechanisms by which bitter compounds open up the airways, the study paves the way for developing safer and more effective therapies for airway obstructive diseases. For one, the results suggest that bitter compounds should be explored as a more effective asthma treatment than currently available L-type calcium channel blockers, which don't work very well. And by simultaneously measuring calcium signaling and cell contraction, scientists will be able to efficiently identify the most promising bitter compounds. Because there are thousands of available bitter substances, some of which can stimulate TAS2Rs at extremely low concentrations, it is likely that very strong bronchodilators can be identified in future studies.

**Zhang C-H, Lifshitz LM, Uy KF, Ikebe M, Fogarty KE, et al. (2013) The Cellular and Molecular Basis of Bitter Tastant-Induced Bronchodilation. doi:10.1371/journal.pbio.1001501**

